# On the Causes and Treatment of Insanity

**Published:** 1855-10-01

**Authors:** D. C. Campbell

**Affiliations:** Physician to the Essex Lunatic Asylum.


### ON THE CAUSES AND TREATMENT OF INSANITY.

BY D. C. CAMPBELL, M.D.,

*Physician to the Essex Lunatic Asylum.*

Religious excitement forms no small number of the assigned causes; and, in accordance with the humane purpose of the institution, I feel it a duty to mention any cause, against the operation of which it may be possible, in some degree, to guard. I do not believe that true religion is ever a cause of insanity, though fanaticism or erroneous theological views undoubtedly may. Mental derangement never can be produced by just views of the essential truths of the Gospel; but intense and long protracted meditation on abstruse points of religious doctrine, or on prophetic mystery, remorse in highly sensitive minds on account of supposed unpardonable sins, and above all, innovation in established religious belief, have been fruitful causes of insanity.

An eminent physician, of great experience in the treatment of mental derangement, in his writings remarked, "Were I to allege one cause which I thought was operating with more force than another to increase the victims of insanity, I should pronounce that it was the overweening zeal with which it is attempted to impress on youth the subtle distinctions of theology and an unrelenting devotion to a dubious doctrine. This practice is an alarming error. It is growing to an excess fatal to the preservation of intellectual sanity, and in a manner especially dangerous to the rising generation." I would recommend to parents to use their best efforts against the influence of new and questionable religious doctrines. The mental distress occasioned by the conflict between such doctrines and earlier religious impressions ends often in confirmed maniacal melancholy; or, as there is a tendency to reaction in our moral as well as in our physical nature, I have seen a sudden transition from the deepest self-abasement to triumphant confidence, with belief in supernatural communications, miraculous gifts, and all the phantasies of an insane mind. Such madness is lamentable in itself; but, in some instances, doubly lamentable, when the patient awakes from his delusion. His religious opinions are then unsettled, and it would be well if he could return to the consolations of that quiet and soothing faith which has given peace to christians in all ages.

Employment in the open air not only improves bodily health, but also powerfully co-operates, with other means of regulating the mind, in promoting the cure of lunacy. Many of the male patients have laboured most assiduously on the farm and in the garden, from which no small saving to the county has arisen; four large airing courts have been laid out by them, which are now finished, and a large garden has been brought into cultivation, now supplying sufficient vegetables for the establishment. Two of the patients are daily employed as carpenters, four as shoemakers, and two assist the engineer and smith. It will be seen by the tables that a considerable quantity of profitable work has also been done by the females, who in addition to that necessary for the establishment, have made a number of shirts for the Springfield prison, and also for a house in London. The females are principally employed in washing, dressing, sewing, and knitting, and a large number of males and females give their assistance in the wards and at domestic work. In several instances I have remarked that the cases were retrograde or progressive, according as the patients were idle or employed. Among those patients who laboured daily, not a few proceeded with a steady pace to recovery, until soundness of mind was perfectly restored.

It requires no proficiency in the study of mind, nor any experience in the treatment of the insane, to comprehend the utility of labour in promoting the cure of lunacy.. Any occupation which serves to arrest the attention of the lunatic necessarily arouses him from his waking dream; and the repose induced by toil no less effectually excludes the visions of the night.

The great object is to make the necessary arrangements of the institution available for the treatment of the patients, to secure as much occupation as possible for them consistent with their health, and to render their services as far as possible advantageous to the institution. The amusements consist of bagatelle, cards, draughts, and dominoes; and books are provided for those who are disposed to read. Several derive much enjoyment from variety of scene, and are permitted to make little excursions into the country, and entertainments with dancing and vocal and instrumental music have been found very serviceable.

The treatment of insanity in all its forms consists less in the administration of medicine than in surrounding the patients with influences, each of which may apparently be very trifling, nay, unfelt and unseen, but the combination of the whole of which produces the most powerful effect; thus they are permitted to enjoy the greatest possible degree of liberty, consistent with their safety, and furnished with the means of such suitable employment, amusements, and recreations, as serve both to relieve the irksomeness of confinement and to promote the cure of the malady. I cannot omit this opportunity of pressing upon public attention the importance of early medical treatment, and the unhappy consequences which arise from the delay so frequently prompted by mistaken affection and shortsighted economy. It is at the commencement of the disease that medical treatment is most obviously demanded and most likely to be efficacious; for notwithstanding the most violent symptoms, if the disorder is of recent occurrence, it generally yields to proper treatment. This important truth, which prejudice, ignorance, and false delicacy are too apt to overlook, is forcibly illustrated in the experience of every asylum. Nothing is more imprudent of the friends of such individuals in trusting, for any period, an unfortunate relative to their own inexperienced, and too often injudicious management, for it is one of the most melancholy attendants on this disease, that it frequently destroys and disregards the ties of nature, and that a patient never can be more unhappily placed than in the circle of his own dearest friends and relatives.—*From the last Report of the Asylum.*

